# PLP-Dependent Enzymes

**DOI:** 10.1155/2014/856076

**Published:** 2014-01-15

**Authors:** Alessandro Paiardini, Roberto Contestabile, Ashley M. Buckle, Barbara Cellini

**Affiliations:** ^1^Dipartimento di Scienze Biochimiche, Sapienza Università di Roma, 00185 Roma, Italy; ^2^Department of Biochemistry and Molecular Biology, Monash University, Clayton, VIC 3800, Australia; ^3^Section of Biological Chemistry, Department of Life Sciences and Reproduction, University of Verona, Strada Le Grazie 8, 37134 Verona, Italy

The main focus of this special issue is on structural, functional, and biomedical studies on pyridoxal-5′-phosphate- (PLP-) dependent enzymes. The unparalleled catalytic versatility of PLP, the active form of vitamin B6, originates from its unique electron-sinking properties, which stabilize reaction intermediates, thus lowering the activation barrier during catalysis. At least five different protein scaffolds arose during evolution to bind PLP and harness its catalytic functionality. The role of the apoenzyme scaffolds is to assist in the proper orientation of the substrate's reacting groups relative to the *π*-electrons of PLP, to promote reactivity and control reaction specificity. In addition, the active site residues interacting with the leaving groups provide either stabilizing or destabilizing interactions to direct catalysis [1].

As a consequence, PLP-dependent enzymes are unrivaled in the variety of reactions they catalyze and the highly diverse metabolic pathways they are involved in, including the conversion of amino acids, one-carbon units, biogenic amines, tetrapyrrolic compounds, and amino sugars. These biocatalysts play also a key role in sulfur assimilation and incorporation in cysteine, biotin, and S-adenosyl methionine.

The consequence of their widespread occurrence and crucial importance is that a number of them are current drug targets. For example, inhibitors of *γ*-aminobutyric acid aminotransferase are used in the treatment of epilepsy [2], serine hydroxymethyltransferase has been identified as a target for cancer therapy [3], and inhibitors of L-DOPA decarboxylase are used in the treatment of Parkinson's disease [4]. Genetic defects affecting PLP enzymes have been also implicated in a number of diseases, including Primary hyperoxaluria type 1, which is caused by mutations in alanine-glyoxylate aminotransferase [5, 6]. Finally, several PLP enzymes are autoantigens in autoimmune disease, for example, glutamate decarboxylase in type I diabetes [7] and SLA/LP in autoimmune hepatitis [8].

This special issue is therefore devoted to the unique and intriguing features of this group of enzymes. Detailed biochemical characterizations of several members of this clan, for example, C-S lyase, glutamate-1-semialdehyde aminomutase, serine hydroxymethyltransferase, and L-DOPA decarboxylase, are described. An original paper, describing the impact of pathogenic mutations of the enzyme serine palmitoyltransferase on its structure and activity, is also provided. Moreover, a review focusing on the role of alanine-glyoxylate aminotransferase homeostasis in the basic mechanisms of primary hyperoxaluria is included. Recent developments and ideas in the field of PLP-dependent enzymes, with a special emphasis given to applied aspects of this research area, have been summarized. The new insights coming from these studies will be hopefully translated into clinically useful agents for innovative therapies to counteract diseases involving PLP enzymes.

*Alessandro Paiardini* *Roberto Contestabile* *Ashley M. Buckle* *Barbara Cellini*

## References

[1] Eliot AC, Kirsch JF (2004). Pyridoxal phosphate enzymes: mechanistic, structural, and evolutionary considerations. *Annual Review of Biochemistry*.

[2] Sarup A, Larsson OM, Schousboe A (2003). GABA transporters and GABA-transaminase as drug targets. *Current Drug Targets: CNS & Neurological Disorders*.

[3] Daidone F, Florio R, Rinaldo S (2011). In silico and in vitro validation of serine hydroxymethyltransferase as a chemotherapeutic target of the antifolate drug pemetrexed. *European Journal of Medicinal Chemistry*.

[4] Daidone F, Montioli R, Paiardini A (2012). Identification by virtual screening and in vitro testing of human DOPA decarboxylase inhibitors. *PLoS ONE*.

[5] Oppici E, Fodor K, Paiardini A (2013). Crystal structure of the S187F variant of human liver alanine:aminotransferase associated with primary hyperoxaluria type I and its functional implications. *Proteins*.

[6] Oppici E, Roncador A, Montioli R, Bianconi S, Cellini B (2013). Gly161 mutations associated with primary hyperoxaluria type I induce the cytosolic aggregation and the intracellular degradation of the apo-form of alanine:glyoxylate aminotransferase. *Biochimica et Biophysica Acta*.

[7] Fenalti G, Buckle AM (2010). Structural biology of the GAD autoantigen. *Autoimmunity Reviews*.

[8] Paiardini A, Pascarella S (2013). Structural mimicry between SLA/LP and Rickettsia surface antigens as a driver of autoimmune hepatitis: insights from an in silico study. *Theoretical Biology and Medical Modelling*.

